# Expression of podocalyxin-like protein is an independent prognostic biomarker in resected esophageal and gastric adenocarcinoma

**DOI:** 10.1186/s12907-016-0034-8

**Published:** 2016-07-29

**Authors:** David Borg, Charlotta Hedner, Björn Nodin, Anna Larsson, Anders Johnsson, Jakob Eberhard, Karin Jirström

**Affiliations:** Department of Clinical Sciences Lund, Division of Oncology and Pathology, Lund University, Skåne University Hospital, SE-221 85 Lund, Sweden

**Keywords:** Esophageal neoplasms, Stomach neoplasms, Adenocarcinoma, Prognosis, *PODXL*

## Abstract

**Background:**

Podocalyxin-like protein (PODXL) is a cell surface transmembrane glycoprotein, the expression of which has been associated with poor prognosis in a range of malignancies. The aim of this study was to investigate the impact of PODXL expression on survival in esophageal and gastric adenocarcinoma.

**Methods:**

The study cohort consists of a consecutive series of 174 patients with esophageal (including the gastroesophageal junction) or gastric adenocarcinoma, surgically treated between 2006 and 2010 and not subjected to neoadjuvant treatment. Immunohistochemical expression of PODXL was assessed in tissue microarrays with cores from primary tumors, lymph node metastases, intestinal metaplasia and adjacent normal epithelium. Survival analyses were performed on patients with no distant metastases and no macroscopic residual tumor.

**Results:**

In the majority of cases, expression of PODXL was significantly higher in cancer cells compared to normal epithelial cells and was significantly associated with lymph node metastases and high grade tumors. In esophageal adenocarcinoma, Kaplan-Meier analyses revealed that patients with PODXL negative tumors had a superior time to recurrence (TTR) and overall survival (OS) compared to patients with PODXL positive tumors. In gastric adenocarcinoma, patients with PODXL negative tumors had a superior TTR and a trend towards an improved OS. In esophageal and gastric adenocarcinoma combined, the prognostic significance of PODXL expression on TTR was confirmed in unadjusted Cox regression analysis (HR = 5.36, 95 % CI 1.68-17.06, *p* = 0.005) and remained significant in the adjusted model (HR = 3.39, 95 % CI 1.01-11.35, *p* = 0.048). Moreover, the impact of PODXL expression on OS was also confirmed in unadjusted analysis (HR = 2.52, 95 % CI 1.31-4.85, *p* = 0.006) and remained significant in the adjusted model (HR = 2.03, 95 % CI 1.04-3.98, *p* = 0.039).

**Conclusions:**

In esophageal and gastric adenocarcinoma, PODXL expression is an independent prognostic biomarker for reduced time to recurrence and poor overall survival. This is the first report on the prognostic role of PODXL in esophageal adenocarcinoma and validates recent findings in gastric cancer.

**Electronic supplementary material:**

The online version of this article (doi:10.1186/s12907-016-0034-8) contains supplementary material, which is available to authorized users.

## Background

Esophageal and gastric cancers are among the most common types of cancer worldwide in terms of incidence and mortality [1]. Historically, the majority of esophageal cancers were squamous cell carcinomas, but in the last four decades there has been a drastic increase in the incidence of adenocarcinoma, especially in many Western countries, where it is now the most common subtype [2]. Adenocarcinoma in the esophagogastric (EG) junction is, since the 7th edition of the AJCC/UICC TNM staging system [3], classified as esophageal cancer. Proposed risk factors for esophageal and EG junction adenocarcinoma are gastroesophageal reflux disease, obesity and decreased prevalence of *Helicobacter pylori* infection [4, 5]. Regarding gastric adenocarcinoma, the incidence has been declining for several decades [6], possibly due to improved sanitary conditions and decreased prevalence of *Helicobacter pylori* infection [7], but globally it is still the 3rd leading cause of cancer death.

In resectable esophageal and gastric cancer, several phase III trials [8–13] have shown that the addition of neoadjuvant and/or adjuvant chemotherapy or chemoradiotherapy improves survival. However, the prognosis is still poor, especially in Western populations, with 5-year survival rates less than 40 %.

Hence, in addition to primary prevention and earlier detection, the key to improved outcome for patients with esophageal and gastric cancer is to find more effective treatments and also to personalize the treatment based on prognostic and response predictive factors.

Podocalyxin-like protein (PODXL) is a cell surface transmembrane glycoprotein, belonging to the CD34 family, that is encoded on chromosome 7q32-q33. It was first discovered in renal podocytes as an anti-adhesive protein [14] and has later been shown to be expressed in vascular endothelium [15] and to be involved in hematopoiesis [16] and neural development [17]. PODXL is expressed in a range of malignancies and overexpression has mostly been linked to poor prognosis, e.g. in glioblastoma multiforme [18], breast cancer [19], bladder cancer [20], periampullary and pancreatic adenocarcinoma [21, 22] and colorectal cancer [23–25]. Laitinen et al. [26] recently showed that in surgically treated gastric cancer, patients with PODXL negative tumors had a significantly better cancer-specific 5-year survival than patients with PODXL positive tumors.

The functional role of PODXL in tumorigenesis is largely unknown, but it has been demonstrated to promote cancer cell invasion and migration and to enhance metastatic potential [27–29]. Other proposed mechanisms are evasion of natural killer cell-mediated cytotoxicity [30] and maintaining and regulating the surface expression of glucose transporters [31]. In osteosarcoma cell lines, PODXL has been shown to promote chemoresistance to cisplatin [32], which is particularly interesting since platinum compounds (cisplatin and oxaliplatin) are important cytotoxic drugs in the treatment of esophageal and gastric adenocarcinoma.

To our best knowledge, there are no reports on the prognostic value of PODXL expression in esophageal adenocarcinoma.

The aim of this study was to explore the expression of PODXL in both esophageal and gastric adenocarcinoma and to assess its impact on time to recurrence (TTR) and overall survival (OS) in a consecutive series of patients from southern Sweden, treated surgically between 2006 and 2010, prior to the wide implementation of neoadjuvant treatment.

## Methods

### Study design and participants

The study cohort consists of a consecutive series of 174 patients with chemo-/radiotherapy-naive esophageal (including EG junction) or gastric adenocarcinoma, subjected to surgical resection at the University Hospitals of Lund and Malmö between January 1, 2006 and December 31, 2010. This cohort has been examined in several previous biomarker studies [33–37]. Data on survival and recurrence were updated until December 31, 2014. Tumor location was based on endoscopy findings. Classification of tumor stage was done according to the 7th edition of the UICC/AJCC TNM classification [3]. Histotype according to Laurén [38] was denoted for all tumors as intestinal, mixed or diffuse growth pattern. This classification is generally applied on gastric cancer but can be used also for esophageal and EG junction adenocarcinoma. Residual tumor status was classified as: R0 = no residual tumor (free resection margins according to pathology report), R1 = possible microscopic residual tumor (narrow or compromised resection margins according to pathology report), R2 = macroscopic residual tumor (according to surgery report). The vast majority (98.7 %) of the patients were operated on with a curative intent but three patients with known distant metastases (M1-disease) were resected to palliate symptoms from the primary tumor. In 16 patients, M1-disease was revealed either during surgery or in the resected specimens. No patients received neoadjuvant oncological therapy and only a minority (7.5 %) of the patients received adjuvant treatment (chemo-/radiotherapy). Clinical data, recurrence status and vital status were obtained retrospectively from medical records. Clinicopathological factors and follow-up data are described in Additional file 1.

### Tissue microarrays

Tissue microarrays (TMAs) were constructed using a semi-automated arraying device (TMArrayer™, Pathology Devices, Westminster, MD, USA). From all 174 primary tumors, duplicate cores (1 mm) from separate donor blocks were obtained. In 81 cases lymph node metastases were sampled in duplicate cores (each from a separate metastasis if more than one). In addition 1–3 cores from areas with intestinal metaplasia (Barrett’s esophagus or gastric intestinal metaplasia) were sampled in 73 cases. Single core samples from adjacent normal esophageal squamous epithelium (96 cases) and normal gastric columnar epithelium (131 cases) were also retrieved. All samples were paired.

### Immunohistochemistry

For immunohistochemical analysis of PODXL expression, 4 μm TMA-sections were automatically pre-treated using the PT Link system and then stained in an Autostainer Plus (DAKO; Glostrup, Copenhagen, Denmark) with the rabbit polyclonal anti-PODXL antibody HPA002110 (Atlas Antibodies AB, Stockholm, Sweden) diluted 1:250. The same antibody was used by Laitinen et al. in their study on gastric cancer [26], and the specificity of the antibody has been validated previously [39]. Staining was assessed simultaneously by two different observers (KJ and DB) blinded to clinical and outcome data and scoring discrepancies were discussed to reach consensus. As in previous studies from our group [20, 21, 24, 25, 40], assessment of PODXL staining was registered as negative (0), weak cytoplasmic positivity in any proportion of cells (1), moderate cytoplasmic positivity in any proportion of cells (2), distinct membranous positivity in ≤50 % of cells (3) and distinct membranous positivity in >50 % of cells (4). For samples with duplicate cores the highest staining score was used.

### Statistical analysis

For description of the cohort and in the analyses of the association between PODXL expression and clinicopathological factors, chi-square test (Fisher’s exact for 2x2 tables and linear-by-linear association for tables with more than two rows) was used for categorical variables and Mann–Whitney *U* test for continuous variables. The Mann–Whitney *U* test was used to assess differences in PODXL expression between tissue types. In the analyses of the association between PODXL expression and clinicopathological factors and in the survival analyses, a dichotomized variable of negative (staining score 0) vs. positive (staining score 1–4) PODXL expression in the primary tumor and/or lymph node metastases was applied. The cut-off between negative and positive PODXL expression was the same as used in the study by Laitinen et al. in gastric cancer [26]. TTR was defined as time from diagnosis (date of result of the preoperative biopsy) to the date of biopsy or radiology proven recurrent disease. OS was defined as time from diagnosis (date of result of the preoperative biopsy) to the date of death. TTR and OS were analyzed for resected patients with M0-disease and no macroscopic residual tumor (R0-1). Differences in Kaplan-Meier survival curves were computed using log-rank test. Unadjusted and adjusted hazard ratios (HR) for survival were determined using Cox proportional-hazards regression. For TTR we adjusted for T stage, N stage, R classification, differentiation grade and adjuvant treatment. For OS, the adjusted model included age, T stage, N stage, R classification and differentiation grade. All tests were 2-sided and a *p*-value <0.05 was considered significant. IBM® SPSS® Statistics version 22.0.0.1 for Mac was used for all statistical analyses.

## Results

### PODXL expression in normal epithelium, intestinal metaplasia, primary tumors and lymph node metastases

**Fig. 1:**
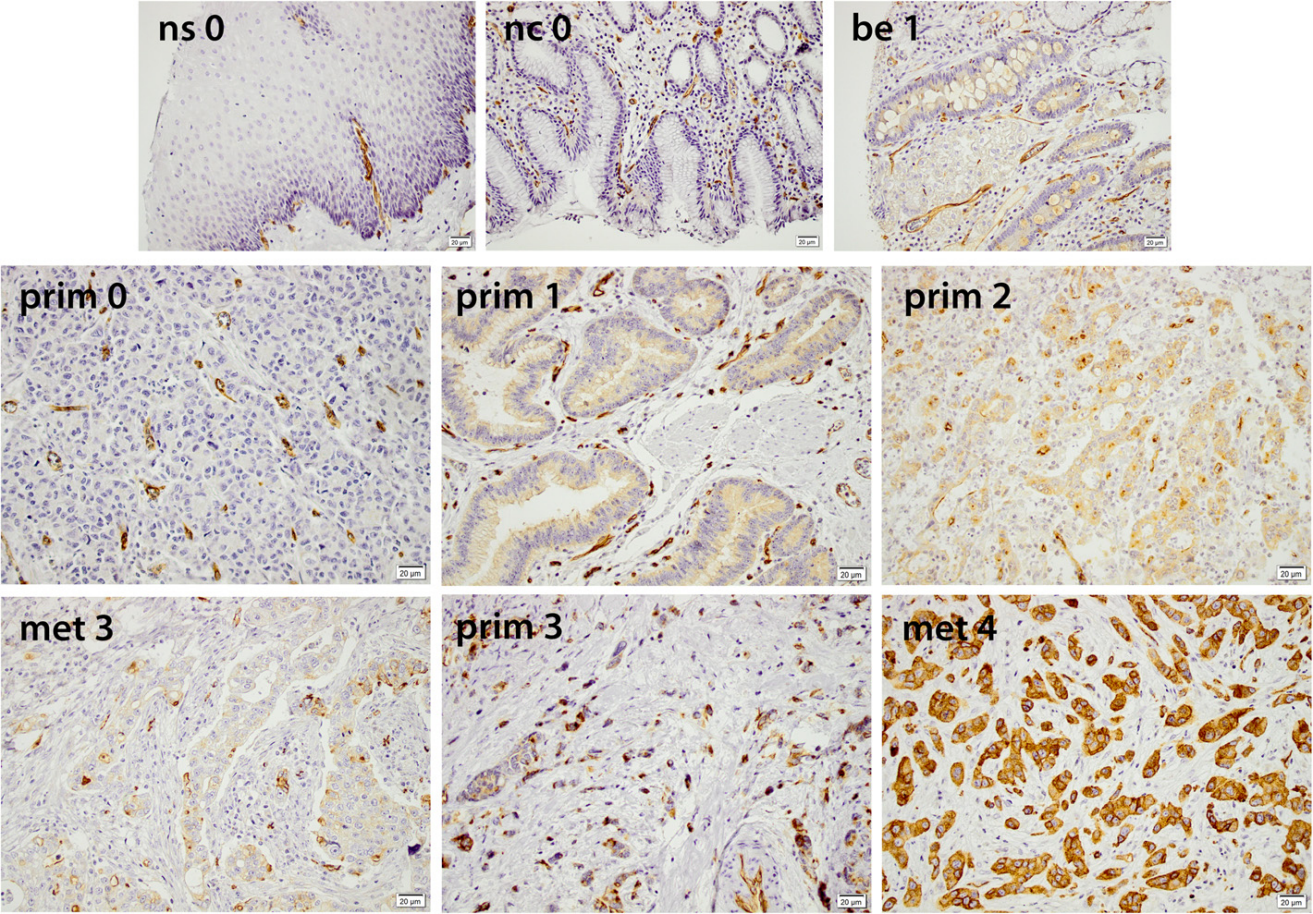
Sample immunohistochemical images of PODXL expression (staining score 0–4), magnification x 20. *Top panel, from left*: Normal squamous epithelium (0). Normal columnar epithelium (0). Barrett’s esophagus (1). *Middle panel, from left*: Primary gastric adenocarcinoma, diffuse subtype (0). Primary gastric adenocarcinoma, intestinal subtype (1). Primary gastric adenocarcinoma, intestinal subtype (2). *Bottom panel, from left*: Lymph node metastasis, intestinal subtype (3). Primary esophageal adenocarcinoma, diffuse subtype (3). Lymph node metastasis, intestinal subtype (4)

**Fig. 2:**
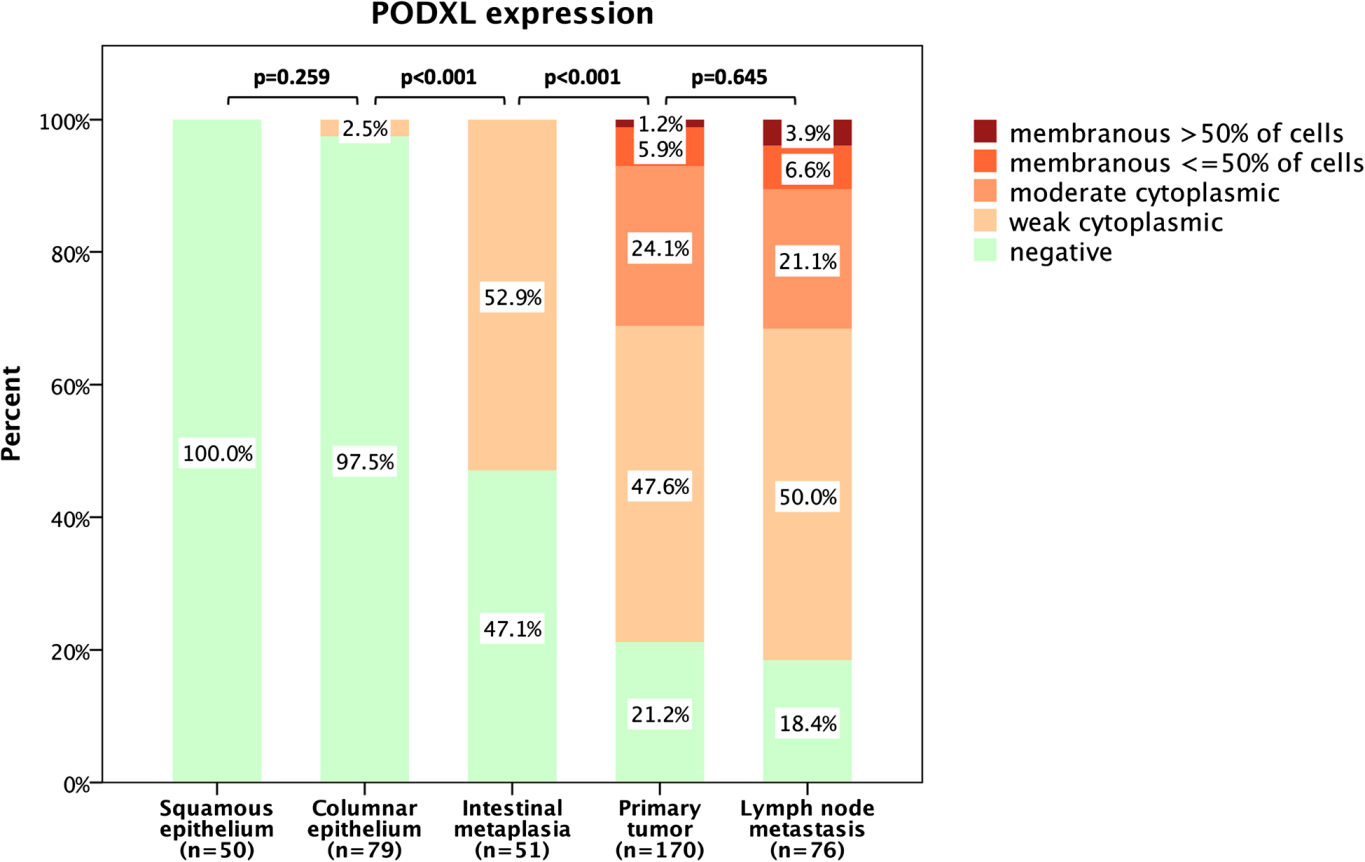
Box plots visualizing the distribution of immunohistochemical expression of PODXL in normal squamous epithelium, normal gastric mucosa, intestinal metaplasia (Barrett’s esophagus or gastric intestinal metaplasia), primary tumors and lymph node metastases in the entire cohort

Immunohistochemical expression of PODXL could be assessed in 50/96 (52 %) samples with normal esophageal squamous epithelium, 79/131 (60 %) samples with normal gastric columnar epithelium, 51/73 (70 %) samples with intestinal metaplasia (Barrett’s esophagus or gastric intestinal metaplasia), 170/174 (98 %) samples with primary tumors, and 76/81 (94 %) samples with lymph node metastases. PODXL staining was mainly detected in the cytoplasm, sometimes in a granular pattern, with or without an accentuation towards the membrane, and in some cases with a strong membranous component. Sample images are shown in Fig. 1. As expected there was a strong staining in endothelial cells, thus serving as an internal positive control. The distribution of immunohistochemical expression of PODXL in the different tissue types is shown in Fig. 2. Expression of PODXL was significantly higher in intestinal metaplasia (Barrett’s esophagus or gastric intestinal metaplasia) compared to normal epithelium (*p* < 0.001) and PODXL expression was significantly higher in primary tumors and lymph node metastases compared to intestinal metaplasia (*p* < 0.001). PODXL expression was similar in Barrett’s esophagus and gastric intestinal metaplasia (*p* = 0.671, data not shown). There was no significant difference in PODXL expression between primary tumors and lymph node metastases (*p* = 0.645) and PODXL expression in primary tumors and/or lymph node metastases did not differ significantly by primary tumor location (*p* = 0.314, data not shown).

### Associations of PODXL expression with clinicopathological factors

As shown in Table 1, factors significantly associated with PODXL expression were N stage and high grade tumors. Negative PODXL expression was denoted in 14.2 % of the esophageal cancers and in 21.5 % of the gastric cancers, but the difference between locations was not statistically significant.

**Table 1:** Associations of PODXL expression with clinicopathological factors

	Entire cohort	PODXL neg	PODXL pos	
	n (%)	n (%)	n (%)	*p-*value
	171 (100.0)	29 (17.0)	142 (83.0)	
Age				
≤70	85 (49.7)	12 (41.4)	73 (51.4)	0.416
>70	86 (50.3)	17 (58.6)	69 (48.6)	
Sex				
Female	39 (22.8)	9 (31.0)	30 (21.1)	0.330
Male	132 (77.2)	20 (69.0)	112 (78.9)	
T stage				
T1	18 (10.5)	2 (6.9)	16 (11.3)	0.637
T2	32 (18.7)	6 (20.7)	26 (18.3)	
T3	94 (55.0)	16 (55.2)	78 (54.9)	
T4	27 (15.8)	5 (17.2)	22 (15.5)	
N stage				
N0	57 (33.3)	16 (55.2)	41 (28.9)	0.006
N1	29 (17.0)	4 (13.8)	25 (17.6)	
N2	41 (24.0)	6 (20.7)	35 (24.6)	
N3	44 (25.7)	3 (10.3)	41 (28.9)	
M stage				
M0	152 (88.9)	25 (86.2)	127 (89.4)	0.535
M1	19 (11.1)	4 (13.8)	15 (10.6)	
R classification				
R0	117 (68.4)	22 (75.9)	95 (66.9)	0.556
R1	45 (26.3)	5 (17.2)	40 (28.2)	
R2	9 (5.3)	2 (6.9)	7 (4.9)	
Differentiation grade				
Low grade	8 (4.7)	4 (13.8)	4 (2.8)	0.023
Intermediate grade	52 (30.4)	10 (34.5)	42 (29.6)	
High grade	111 (64.9)	15 (51.7)	96 (67.6)	
Lauren classification				
Intestinal	119 (69.6)	23 (79.3)	96 (67.6)	0.335
Mixed	9 (5.3)	0 (0.0)	9 (6.3)	
Diffuse	43 (25.1)	6 (20.7)	37 (26.1)	
Location				
Esophagus + EG junction	106 (62.0)	15 (51.7)	91 (64.1)	0.217
Stomach	65 (38.0)	14 (48.3)	51 (35.9)	

R0 = no residual tumor (free resection margins according to pathology report), R1 = possible microscopic residual tumor (narrow or compromised resection margins according to pathology report), R2 = macroscopic residual tumor (according to surgery report)

N1 = metastasis in 1–2 regional lymph nodes, N2 = metastasis in 3–6 regional lymph nodes, N3 = metastasis in 7 or more regional lymph nodes

The dichotomized variable for age is based on the mean/median age, as shown in Additional file 1

### Impact of PODXL expression on prognosis

**Fig. 3:**
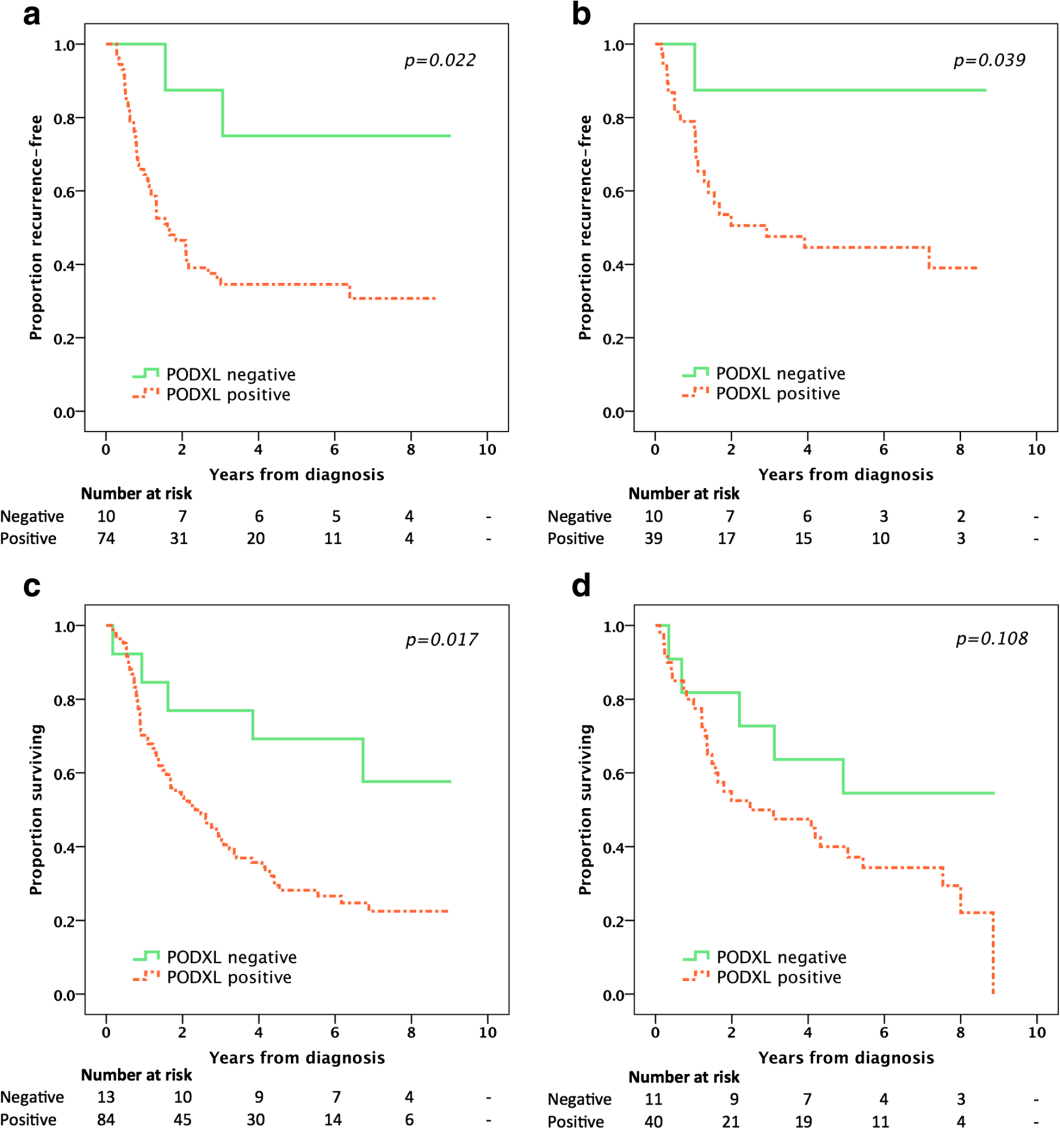
Kaplan-Meier plots of time to recurrence and overall survival according to PODXL expression in patients with M0-disease and no macroscopic residual tumor (R0-1). Time to recurrence in **a** esophageal cancer and **b** gastric cancer. Overall survival in **c** esophageal cancer and **d** gastric cancer

Survival analyses were performed on patients with M0-disease and no macroscopic residual tumor (R0-1). In esophageal adenocarcinoma the Kaplan-Meier analyses (Fig. 3a, c) revealed that patients with PODXL negative tumors had a superior TTR (estimated recurrence-free rate at 5 years 75 % vs. 35 %) and OS (estimated surviving rate at 5 years 69 % vs. 28 %) compared to patients with PODXL positive tumors. In gastric adenocarcinoma, patients with PODXL negative tumors had a superior TTR (estimated recurrence-free rate at 5 years 88 % vs. 45 %) and a trend towards an improved OS (estimated surviving rate at 5 years 55 % vs. 40 %) in the Kaplan-Meier analyses (Fig. 3b, d). In esophageal and gastric adenocarcinoma combined, as shown in Table 2, the prognostic significance of PODXL expression on TTR was confirmed in unadjusted Cox regression analysis (HR = 5.36, 95 % CI 1.68-17.06, *p* = 0.005) and remained significant in the adjusted model (HR = 3.39, 95 % CI 1.01-11.35, *p* = 0.048). Moreover, the impact of PODXL expression on OS was also confirmed in unadjusted analysis (HR = 2.52, 95 % CI 1.31-4.85, *p* = 0.006) and remained significant in the adjusted model (HR = 2.03, 95 % CI 1.04-3.98, *p* = 0.039).

**Table 2:** Hazard ratios for recurrence and death (M0, R0-1)

	Time to recurrence					Overall survival				
		Unadjusted		Adjusted			Unadjusted		Adjusted	
	*n* (events)	HR (95 % CI)	*p-value*	HR (95 % CI)	*p-value*	*n* (events)	HR (95 % CI)	*p-value*	HR (95 % CI)	*p-*value
Age										
Continuous	136 (72)	1.00 (0.98–1.02)	0.610			151 (104)	1.04 (1.02–1.06)	<0.001	1.05 (1.03–1.07)	<0.001
Sex										
Female	27 (12)					30 (22)				
Male	109 (60)	1.18 (0.63–2.19)	0.610			121 (82)	0.81 (0.50–1.29)	0.370		
T stage			<0.001		0.043			0.010		0.511
T1	18 (3)					19 (7)				
T2	27 (8)	2.14 (0.57–8.06)	0.263	1.53 (0.39–5.96)	0.538	30 (19)	2.09 (0.88–4.97)	0.097	1.53 (0.62–3.78)	0.357
T3	77 (51)	6.92 (2.15–22.29)	0.001	3.72 (1.10–12.64)	0.035	86 (66)	3.27 (1.49–7.15)	0.003	1.79 (0.79–4.08)	0.164
T4	14 (10)	8.52 (2.33–31.16)	0.001	3.52 (0.92–13.56)	0.067	16 (12)	3.71 (1.45–9.48)	0.006	1.98 (0.74–5.31)	0.175
N stage										
N0	50 (7)					53 (26)				
N1-3	86 (65)	9.89 (4.51–21.72)	<0.001	7.78 (3.24–18.71)	<0.001	98 (78)	2.56 (1.63–4.00)	<0.001	2.79 (1.67–4.66)	<0.001
R classification										
R0	103 (46)					113 (69)				
R1	33 (26)	2.89 (1.76–4.74)	<0.001	1.38 (0.79–2.41)	0.253	38 (35)	2.75 (1.80–4.20)	<0.001	2.07 (1.29–3.31)	0.003
Differentiation grade										
Low/Intermediate grade	49 (18)					56 (31)				
High grade	87 (54)	2.22 (1.30–3.79)	0.004	1.93 (1.09–3.42)	0.025	95 (73)	1.73 (1.13–2.63)	0.011	1.40 (0.88–2.20)	0.153
Lauren classification										
Intestinal	94 (45)					106 (69)				
Diffuse/Mixed	42 (27)	1.57 (0.97–2.53)	0.064			45 (35)	1.35 (0.90–2.03)	0.149		
Adjuvant treatment										
No	126 (63)					138 (94)				
Chemo-/radiotherapy	10 (9)	2.12 (1.05–4.28)	0.036	0.96 (0.45–2.06)	0.924	13 (10)	1.20 (0.62–2.30)	0.589		
Location										
Esophagus + EG junction	86 (49)					99 (69)				
Stomach	50 (23)	0.74 (0.45–1.22)	0.239			52 (35)	0.90 (0.60–1.34)	0.592		
PODXL expression										
Negative	20 (3)					24 (10)				
Positive	113 (67)	5.36 (1.68–17.06)	0.005	3.39 (1.01–11.35)	0.048	124 (92)	2.52 (1.31–4.85)	0.006	2.03 (1.04–3.98)	0.039

R0 = no residual tumor (free resection margins according to pathology report), R1 = possible microscopic residual tumor (narrow or compromised resection margins according to pathology report)

Similar results were obtained when the survival analyses were stratified by primary tumor location (Additional file 2), and when considering PODXL expression in primary tumors and lymph node metastases separately (Additional file 3).

## Discussion

In this study on resected esophageal and gastric adenocarcinoma we have shown that PODXL is expressed in the majority of cases and correlates with poor survival, but in the subgroup of patients with PODXL negative cancers the prognosis was excellent. This finding applies to both esophageal and gastric cancer with regard to both TTR and OS. To our best knowledge, this is the first report on the prognostic role of PODXL in esophageal adenocarcinoma. Furthermore, in gastric cancer, we have validated the recent findings from Laitinen et al. [26] of a negative prognostic impact of PODXL expression, even though the proportion of PODXL negative gastric cancer cases were lower in our study (21.5 % compared to 42.5 %). The reasons for this discrepancy are not clear, since we used the same polyclonal antibody and the same definition for negative vs. positive PODXL expression. However, whereas Laitinen et al. only examined primary tumors, we also included lymph node metastases in our analyses. This resulted in a non-significant (*p* = 0.506) decrease in PODXL negative gastric cancer cases from 26.6 to 21.5 %. Another factor could be observer-dependent, such as setting the cut-off between negative and weak cytoplasmic staining. In other reports on PODXL as a prognostic marker in colorectal [23–25, 40], pancreatic and periampullary adenocarcinoma [21, 22], using the same polyclonal antibody, the most evident prognostic cut-off was observed for membranous vs. non-membranous expression, with the former being an independent factor of poor prognosis. However, in our study and in the report from Laitinen et al., the optimal prognostic cut-off was negative vs. positive, including membranous, PODXL expression. Hence, further studies are warranted to determine optimal cut-offs for prognostication, which may well differ between different types of cancer. Of note, previous studies on colorectal [41] and pancreatic [22] cancer, using a monoclonal anti-PODXL antibody, demonstrated a correlation between cytoplasmic PODXL expression and poor survival.

A limitation of this study is its retrospective design. However, we have managed to access all the necessary clinical data, except for recurrence status in some cases, and the tissue specimens have been thoroughly re-examined. Due to heterogeneity within tumors there is always a risk of sampling bias with the TMA-technique. However, as we used duplicate cores from different donor blocks and, when available, also included cores from lymph node metastases when denoting the highest PODXL score for each case, the risk of overestimating the proportion of PODXL negative cancers should be reduced.

In current practice, most patients with resectable esophageal or gastric adenocarcinoma receive neoadjuvant and/or adjuvant chemotherapy or chemoradiotherapy, but only a minority (10-15 %) of the patients actually benefit from the oncological treatment [8–13]. For a biomarker to be really useful in clinical decision making it should not only be prognostic but also be able to predict whether a patient will benefit from a treatment or not. Further studies on PODXL in esophageal and gastric adenocarcinoma are warranted to validate its role as a prognostic biomarker and to explore whether it also may be useful as a treatment response predictive biomarker, as suggested in previous studies on colorectal [24] and periampullary cancer [21].

## Conclusions

In summary, we have shown that PODXL is commonly expressed in esophageal and gastric adenocarcinoma and associated with lymph node metastases and high grade tumors. Furthermore, PODXL is an independent prognostic biomarker for reduced time to recurrence and poor overall survival, but in the subgroup of patients with PODXL negative tumors the prognosis appears to be excellent.

## Abbreviations

CI, confidence interval; EG, esophagogastric; HR, hazard ratio; IHC, immunohistochemistry; OS, overall survival; PODXL, podocalyxin-like protein; TMA, tissue microarray; TTR, time to recurrence

## Additional files

Additional file 1: Table S1. Detailed description of the cohort. (DOCX 19 kb) Additional file 2: Table S2. Hazard ratios for recurrence and death stratified by primary tumor location. (DOCX 18 kb) Additional file 3: Table S3. Hazard ratios for recurrence and death stratified by PODXL expression in primary tumors or lymph node metastases (separately and combined). (DOCX 18 kb)
