# Supplementary material for: Expression of podocalyxin-like protein is an independent prognostic biomarker in resected esophageal and gastric adenocarcinoma
Source: BMC Clin Pathol. 2016 Jul 29;16:13. doi: 10.1186/s12907-016-0034-8 (PMC4966733; doi:10.1186/s12907-016-0034-8)
Supplement: Additional file 1: Table S1. — Detailed description of the cohort. (DOCX 19 kb) [file 12907_2016_34_MOESM1_ESM.docx]

| **Additional file** **1: Table S1. Patient and tumor characteristics** | | | | |
| --- | --- | --- | --- | --- |
| **Factor** | **Entire cohort**  (n=174)  n (%) | **Esophagus + EGJ**  (n=108)  n (%) | **Stomach**  (n=66)  n (%) | *p-value* |
| **Age** (years)  Mean  Median  Range | 70.2  70.0  42.6-94.4 | 69.1  68.7  48.2-88.6 | 72.0  73.1  42.6-94.4 | 0.066 |
| **Sex**  Women  Men | 39 (22.4)  135 (77.6) | 17 (15.7)  91 (84.3) | 22 (33.3)  44 (66.7) | **0.009** |
| **T stage**  T1  T2  T3  T4 | 19 (10.9)  32 (18.4)  96 (55.2)  27 (15.5) | 12 (11.1)  15 (13.9)  69 (63.9)  12 (11.1) | 7 (10.6)  17 (25.8)  27 (40.9)  15 (22.7) | 0.954 |
| **N stage**  N0  N1  N2  N3 | 59 (33.9)  30 (17.2)  41 (23.6)  44 (25.3) | 28 (25.9)  20 (18.5)  29 (26.9)  31 (28.7) | 31 (47.0)  10 (15.2)  12 (18.2)  13 (19.7) | **0.011** |
| **Number of examined nodes**  Mean  Median  Range  Unknown | 30  29  1-112  14 | 34  31  8-72  3 | 24  22  1-112  11 | **< 0.001** |
| **M stage**  M0  M1 | 155 (89.1)  19 (10.9) | 100 (92.6)  8 (7.4) | 55 (83.3)  11 (16.7) | 0.079 |
| **R classification**  R0  R1  R2 | 119 (68.4)  46 (26.4)  9 (5.2) | 70 (64.8)  35 (32.4)  3 (2.8) | 49 (74.2)  11 (16.7)  6 (9.1) | 0.732 |
| **Differentiation grade**  Low grade  Intermediate grade  High grade | 8 (4.6)  53 (30.5)  113 (64.9) | 5 (4.6)  40 (37.0)  63 (58.3) | 3 (4.5)  13 (19.7)  50 (75.8) | 0.052 |
| **Lauren classification**  Intestinal  Mixed  Diffuse | 120 (69.0)  9 (5.2)  45 (25.9) | 89 (82.4)  6 (5.6)  13 (12.0) | 31 (47.0)  3 (4.5)  32 (48.5) | **< 0.001** |
| **Adjuvant therapy**  No  Chemoradiotherapy  Chemotherapy  Radiotherapy | 161 (92.5)  11 (6.3)  1 (0.6)  1 (0.6) | 100 (92.6)  6 (5.6)  1 (0.9)  1 (0.9) | 61 (92.4)  5 (7.6) | 0.520 |
| **Follow-up (years)**  Mean  Median  Range | 3.4  2.4  0.1-9.3 | 3.4  2.5  0.2-9.3 | 3.4  2.1  0.1-8.9 | 0.754 |
| **Recurrence**  No  Yes  Unknown/Not applicable | 66 (37.9)  80 (46.0)  28 (16.1) | 38 (35.2)  53 (49.1)  17 (15.7) | 28 (42.4)  27 (40.9)  11 (16.7) | 0.871 |
| **Vital status**  Alive  Dead | 47 (27.0)  127 (73.0) | 30 (27.8)  78 (72.2) | 17 (25.8)  49 (74.2) | 0.861 |
| R0 = no residual tumor (free resection margins according to pathology report), R1 = possible microscopic residual tumor (narrow or compromised resection margins according to pathology report), R2 = macroscopic residual tumor (according to surgery report)  N1 = metastasis in 1–2 regional lymph nodes, N2 = metastasis in 3–6 regional lymph nodes, N3 = metastasis in 7 or more regional lymph nodes | | | | |
