# Supplementary material for: Expression of podocalyxin-like protein is an independent prognostic biomarker in resected esophageal and gastric adenocarcinoma
Source: BMC Clin Pathol. 2016 Jul 29;16:13. doi: 10.1186/s12907-016-0034-8 (PMC4966733; doi:10.1186/s12907-016-0034-8)
Supplement: Additional file 2: Table S2. — Hazard ratios for recurrence and death stratified by primary tumor location. (DOCX 18 kb) [file 12907_2016_34_MOESM2_ESM.docx]

| **Additional file** **2:** **Table S2. Hazard ratios for recurrence and death (M0, R0-1) stratified by primary tumor location** | | | | | | | | | | |
| --- | --- | --- | --- | --- | --- | --- | --- | --- | --- | --- |
|  | **Time to recurrence** | | | | | **Overall survival** | | | | |
|  |  | **Unadjusted** | | **Adjusted^1^** | |  | **Unadjusted** | | **Adjusted^2^** | |
|  | n (events) | HR (95% CI) | *p-value* | HR (95% CI) | *p-value* | n (events) | HR (95% CI) | *p-value* | HR (95% CI) | *p-value* |
| **Esophagus + EG junction**  PODXL negative  PODXL positive | 10 (2)  74 (46) | 4.53 (1.10-18.73) | **0.037** | 2.76 (0.63-12.03) | 0.176 | 13 (5)  84 (63) | 2.90 (1.16-7.24) | **0.022** | 2.30 (0.89-5.95) | 0.086 |
| **Stomach**  PODXL negative  PODXL positive | 10 (1)  39 (21) | 6.33 (0.85-47.10) | 0.072 | 8.75 (0.86-88.76) | 0.066 | 11 (5)  40 (29) | 2.15 (0.83-5.59) | 0.117 | 1.69 (0.57-5.01) | 0.346 |
| **All locations**  PODXL negative  PODXL positive | 20 (3)  113 (67) | 5.36 (1.68-17.06) | **0.005** | 3.39 (1.01-11.35) | **0.048** | 24 (10)  124 (92) | 2.52 (1.31-4.85) | **0.006** | 2.03 (1.04-3.98) | **0.039** |
| 1) Adjusted for: T stage, N stage, R classification, differentiation grade and adjuvant treatment  2) Adjusted for: age, T stage, N stage, R classification and differentiation grade | | | | | | | | | | |
