# Supplementary material for: Expression of podocalyxin-like protein is an independent prognostic biomarker in resected esophageal and gastric adenocarcinoma
Source: BMC Clin Pathol. 2016 Jul 29;16:13. doi: 10.1186/s12907-016-0034-8 (PMC4966733; doi:10.1186/s12907-016-0034-8)
Supplement: Additional file 3: Table S3. — Hazard ratios for recurrence and death stratified by PODXL expression in primary tumors or lymph node metastases (separately and combined). (DOCX 18 kb) [file 12907_2016_34_MOESM3_ESM.docx]

| **Additional file** **3: Table S3 Hazard ratios for recurrence and death (M0, R0-1) stratified by PODXL expression in primary tumors or lymph node metastases (separately and combined)** | | | | | | | | | | |
| --- | --- | --- | --- | --- | --- | --- | --- | --- | --- | --- |
| **PODXL expression** | **Time to recurrence** | | | | | **Overall survival** | | | | |
|  |  | **Unadjusted** | | **Adjusted^1^** | |  | **Unadjusted** | | **Adjusted^2^** | |
|  | n (events) | HR (95% CI) | *p-value* | HR (95% CI) | *p-value* | n (events) | HR (95% CI) | *p-value* | HR (95% CI) | *p-value* |
| **Primary tumor**  negative  positive | 26 (8)  106 (61) | 2.12 (1.02-4.44 | **0.046** | 1.37 (0.62-3.01) | 0.433 | 30 (14)  117 (87) | 2.14 (1.21-3.77) | **0.009** | 1.82 (1.02-3.26) | **0.044** |
| **Lymph node metastases**  negative  positive | 10 (7)  44 (38) | 3.18 (1.39-7.30) | **0.006** | 3.04 (1.15-8.02) | **0.025** | 12 (7)  52 (44) | 2.53 (1.14-5.56) | **0.023** | 3.88 (1.48-10.16) | **0.006** |
| **Primary tumor and lymph node metastases**  negative  positive | 20 (3)  113 (67) | 5.36 (1.68-17.06) | **0.005** | 3.39 (1.01-11.35) | **0.048** | 24 (10)  124 (92) | 2.52 (1.31-4.85) | **0.006** | 2.03 (1.04-3.98) | **0.039** |
| 1) Adjusted for: T stage, N stage, R classification, differentiation grade and adjuvant treatment  2) Adjusted for: age, T stage, N stage, R classification and differentiation grade | | | | | | | | | | |
